# Comparative Effectiveness of Intravenous Immunoglobulin for Children with Kawasaki Disease: A Nationwide Cohort Study

**DOI:** 10.1371/journal.pone.0063399

**Published:** 2013-05-01

**Authors:** Ming-Chih Lin, Yun-Ching Fu, Sheng-Ling Jan, Mei-Shu Lai

**Affiliations:** 1 Department of Pediatrics, Taichung Veterans General Hospital, Taichung, Taiwan; 2 Graduate Institute of Epidemiology and Preventive Medicine, National Taiwan University, Taipei, Taiwan; 3 Department of Pediatrics and Institute of Clinical Medicine, National Yang-Ming University, Taipei, Taiwan; 4 Center of Comparative Cost Effectiveness Research, National Center of Excellence for Clinical Trial and Research, National Taiwan University Hospital, Taipei, Taiwan; National Taiwan University Hospital, Taiwan

## Abstract

**Introduction:**

Different immunoglobulin manufacturing processes may influence its effectiveness for Kawasaki disease. However, nationwide studies with longitudinal follow-up are still lacking. The aim of this study was to evaluate the comparative effectiveness of immunoglobulin preparations from a nationwide perspective.

**Materials and Methods:**

This is a nationwide retrospective cohort study with a new user design. Data came from the National Health Insurance Research Database of Taiwan. From 1997 to 2008, children under 2 years old who received immunoglobulin therapy for the first time under the main diagnosis of Kawasaki disease were enrolled. The manufacturing processes were divided into β-propiolactonation, acidification and those containing IgA. The endpoints were immunoglobulin non-responsiveness, acute aneurysm, prolonged use of anti-platelets or anti-coagulants, and recurrence.

**Results:**

In total, 3830 children were enrolled. β-propiolactonation had a relative risk of 1.45 (95% CI 1.08∼1.94) of immunoglobulin non-responsiveness, however, the relative risks for acidification and containing IgA were non-significant. For acute aneurysms, acidification had a relative risk of 1.49 (95% CI 1.17∼1.90), however the relative risks for β-propiolactonation and containing IgA were non-significant. For prolonged use of anti-platelets or anti-coagulants, β-propiolactonation had a relative risk of 1.44 (95% CI 1.18∼1.76), and acidification protected against them both with a relative risk of 0.82 (95% CI 0.69∼0.97), whereas the relative risk for containing IgA was non-significant. For recurrence, all three factors were non-significant.

**Conclusions:**

The effectiveness of immunoglobulin may differ among different manufacturing processes. β-propiolactonation had a higher risk of treatment failure and prolonged use of anti-platelets or anti-coagulants. Acidification may increase the risk of acute coronary aneurysms.

## Introduction

Kawasaki disease is the most common form of acquired heart disease among children in most industrialized countries [1]–[9]. It is a systemic vasculitis, and predominantly involves the coronary arteries [10]–[15]. It can cause coronary aneurysms in the acute phase [16]–[18], which can lead to long-term sequelae such as coronary stenosis or obstruction [19], [20].

Intravenous immunoglobulin (IVIG) is the most effective therapy for acute Kawasaki disease. The American Heart Association (AHA) and American Academy of Pediatrics (AAP) have published guidelines which suggest rapid infusion of high dose (2 gm/kg) immunoglobulin within 12 hours and concomitant use of aspirin [21]. IVIG therapy has been shown to decrease the rate of coronary aneurysms from 20∼25% to less than 5% [18], [20], [22].

Immunoglobulin is obtained from blood. Although the World Health Organization has published rules for production [23], there are differences in the manufacturing process such as purification, IgA concentration and condition of preservation [24]–[28]. Whether these differences lead to differences in effectiveness has seldom been studied. In 1995, Venoglobulin-I (Alpha Therapeutics, Los Angles, Calif.) and Iveegam (Immuno AG, Vienna, Austria) were compared in a small case control study. The results revealed that Iveegam had a shorter febrile duration and fewer side effects [29]. However, it was a retrospective study with limited case numbers. In the Kawasaki disease guidelines published by the AHA and AAP in 2004, the comparative effectiveness of different preparations of immunoglobulin was noted as inconclusive [21]. After the guidelines were published, however, two studies studied the comparative effectiveness. The first study was a retrospective review from a single institution, and enzyme processing (beta-propriolactonation) was found to be associated with a higher rate of treatment failure [28]. The other study was also a retrospective study from Canadian registry data, and the authors found that preservation in acidic conditions led to a lower rate of treatment failure but a higher rate of coronary aneurysms [26]. However, both studies had a limited number of cases and lacked longitudinal follow-up.

The National Health Insurance (NHI) program was implemented in Taiwan in 1995. It is a compulsory social welfare program and covers more than 98% of the 22 million people in Taiwan [30], [31]. Taiwan has the third highest incidence of Kawasaki disease in the world [3]. The aim of this study was to compare the effectiveness among different immunoglobulin preparations from a nationwide perspective with longitudinal follow-up data in terms of treatment failure, coronary aneurysms and recurrence by analyzing claims data from the National Health Insurance Research Database (NHIRD).

## Materials and Methods

This is a population based retrospective cohort study. Although the NHI program was initiated in 1995, the data in the NHIRD for 1995 and 1996 are incomplete. Consequently, we retrieved the claims data of inpatient expenditure by admissions (DD files), details of inpatient orders (DO files), ambulatory care expenditure by visit (CD files) and details of ambulatory care orders (OO files) in the NHIRD from 1997 to 2008. The registry for contracted medical facilities (HOSB) and registry for beneficiaries (ID) were also acquired.

### Study population

Children under 2 years old who were admitted to hospitals and received immunoglobulin therapy for the first time under the main diagnosis of Kawasaki disease were enrolled. We defined a main diagnosis of Kawasaki disease as the first or second diagnosis code being Kawasaki disease (ICD9-CM (international classification of diseases, 9th revision, clinical modification) 446.1). The children were regarded as having undergone IVIG therapy if their inpatient orders included medications of anatomical therapeutic classification (ATC) code J06BA02. The index day was defined as the day of admission. A total of five brands of immunoglobulin were reimbursed by the NHI during the study period, including Gamimune N (Talecris Biotherapeutics Inc., NC, USA), Intraglobin F (Biotest Pharma GMBH, Germany), Venoglobulin S (Alpha Therapeutic Corp., LA, USA), Flebogamma (Instituto Crifols S.A., Spain) and TBSF human immunoglobulin (CSL Limited, Australia). Patients born before 1997 were excluded because we could not confirm whether or not they were using IVIG for the first time. Because TBSF human immunoglobulin was only reimbursed from 2008, we excluded it from analysis due to the short follow-up time.

### Classification of IVIG

The details of the IVIG brands are summarized in Table 1. The main independent variables included acidification (brand A versus B, C, D), IgA depletion (brand C, D versus A, B) and enzyme processing (beta-propriolactonation) (brand B versus A, C, D).

**Table 1:** The differences between brands of immunoglobulin products.

	Brand A	Brand B	Brand C	Brand D
**Brand Name**	Gamimune N	Intraglobin F	Venoglobulin-S	Flebogamma
**Manufacturer**	Talecris Biotherapeutics, INC.	Biotest Pharma GMBH	Alpha Therapeutic Corp.	Instituto Crifols S.A.
**Country**	NC, U.S.A.	Germany	LA, U.S.A.	Spain
**Preparation**	Cold ethanol-PEG precipitation, diafiltration/acidification	Cold ethanol-PEG precipitation, β-propiolactonation	Cold ethanol-PEG precipitation, DEAE-Sephadex fractionation	Cold ethanol-PEG precipitation, nanofiltration down to 20 nm
**IgA conc. (ug/mL)**	270	< = 2500	15.1	<50
**IgM conc. (ug/mL)**	76+/−15	< = 600	<11.1	trace
**Osmolality (mOsmol/kg)**	274	N/A	N/A	N/A
**pH**	4.0∼4.5	pH 6.6.	Non acidic	5.0–6.0
**Sugar content**	none	glucose	sorbitol	sorbitol
**IgG subclass**				
**IgG 1 (%)**	59.8	62.0	66.5	66.7
**IgG 2 (%)**	28.9	34.0	24.5	28.2
**IgG 3 (%)**	6.2	0.5	5.8	2.3
**IgG 4 (%)**	5.1	3.5	3.2	2.5
**IgG MWD**	Mono 99%, dimer <1%	Mono+dimer>90%	Mono+dimer>95%	Mono>99.8%
**Period (years)**	1997∼2008	1998∼2004	1998∼2006	2004∼2008

### Covariates

The patients' gender, age and hospital level were collected for analysis. The number of febrile days was defined by the prescription of acetaminophen (ATC code N02BE) or non-steroidal anti-inflammatory drugs (NSAID) (ATC code M01A). If the admission diagnostic codes included ICD9-CM 745∼747, the patients were regarded as having congenital heart diseases.

### Primary outcome (IVIG non-responsiveness)

If the patients received two or more courses of IVIG, they were defined as being non-responsive to IVIG therapy. We defined non-responsiveness as the total amount exceeding 30 gm in one admission or readmission for IVIG therapy within 30 days after the index day.

### Secondary outcomes

The patients were regarded as having an acute coronary aneurysm if the diagnostic codes included ICD9-CM 414.11. We defined prolonged use of anti-platelets or anti-coagulants as patients receiving aspirin (ATC code, N02BA) or anti-coagulation agents (ATC code, B01A) between 180 and 360 days after the index day. Recurrence was defined as readmission for IVIG due to Kawasaki disease more than 30 days after the index day.

### Statistical analysis

For binary outcomes including IVIG non-responsiveness and aneurysm formation, crude relative risks and confidence intervals were calculated separately for acidification, IgA depletion and beta-propriolactonation. Multiple logistic regression models were then applied to calculate the adjusted odds ratios. Model 0 represented the crude odds ratio, Model 1 adjusted for febrile duration, hospital level and gender, and Model 2 adjusted for febrile duration, hospital level, gender, congenital heart diseases, age and period (years). Finally, acidification and beta-propriolactonation were put into the same logistic regression model to test whether an interaction existed. For the primary outcome, we also performed sensitivity analysis to test the stability of the outcome measures when the definition of IVIG non-responsiveness was changed from 24 to 38 gm.

For the time-dependent outcomes, the Kaplan-Meier method was used to compare the recurrence-free survival rate. The Cox regression model was then applied to adjust for the possible confounding factors including febrile duration, hospital level, gender, congenital heart diseases, age and period (years).

Data retrieval and data analysis were conducted by SAS version 9.1 for Windows (SAS Institute, Inc., Cary, NC). Survival curves were depicted by SPSS version 13.0 for Windows (SPSS, Inc., Chicago, IL). A *p* level less than 0.05 was defined as being statistically significant.

### Patients' privacy protection

According to the NHIRD, data which could be used to identify patients or care providers, including medical institutions and physicians, are scrambled before being sent to the National Health Research Institutes (NHRI) for database construction, and further scrambled before being released to individual researchers (http://w3.nhri.org.tw/nhird//en/Data_Protection.html). The computer-processed personal data protection law and related regulations of the Bureau of National Health Insurance and the NHRI of Taiwan were strictly followed. The protocol was reviewed and approved by the NHRI before data release. This study protocol was also approved by the Institutional Review Board of Taichung Veterans General Hospital.

## Results

**Figure 1:**
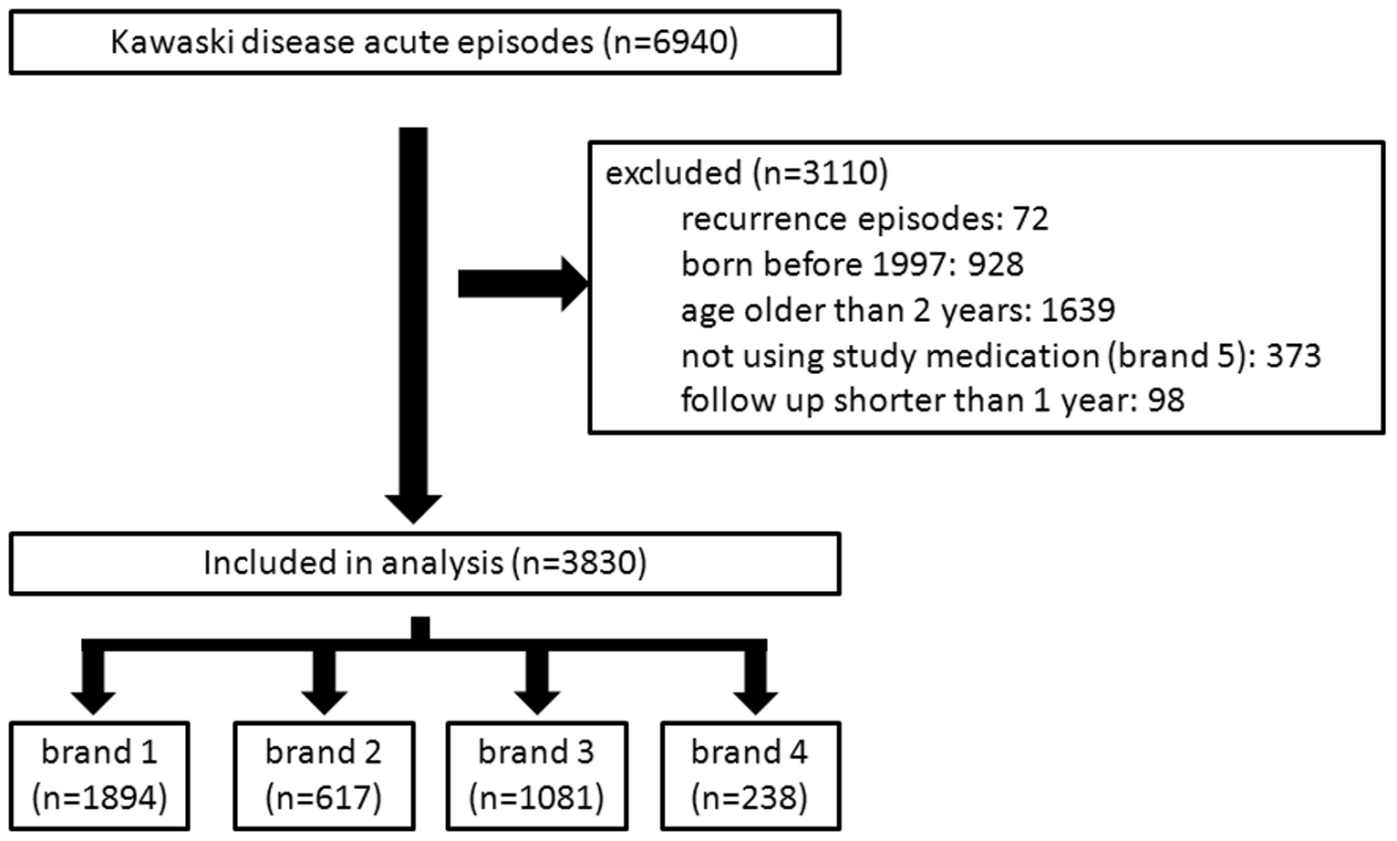
The derivation of the study cohort.

The derivation of the study cohort is summarized in Figure 1. A total of 6940 cases of acute Kawasaki disease episodes were screened, of which 72 recurrent episodes, 928 children born before 1997, 1639 children older than 2 years, 373 episodes not using the study medications, and 98 children with a follow-up time less than 1 year were excluded. In total, 3830 children were enrolled in this study. The demographic data are summarized in Table 2.

**Table 2:** Demographic data for the study patients.

factors		n	%
age group (years)	0∼1	2285	59.66%
	1∼2	1545	40.34%
	Total	3830	100.00%
gender	girls	1412	36.87%
	boys	2412	62.98%
	missing	6	0.16%
	Total	3830	100.00%
hospital level	medical center	2487	64.93%
	regional hospital	1343	35.07%
	Total	3830	100.00%
congenital heart disease	No	3714	96.97%
	Yes	116	3.03%
	Total	3830	100.00%
acute aneurysm	No	3577	93.39%
	Yes	253	6.61%
	Total	3830	100.00%
prolonged use of anti-platelet or anti-coagulant	No	3364	87.83%
	Yes	466	12.17%
	Total	3830	100.00%
recurrence	No	3771	98.46%
	Yes	59	1.54%
	Total	3830	100.00%
days of fever	No records	620	16.19%
	0∼3 days	299	7.81%
	3∼6 days	384	10.03%
	6∼9 days	169	4.41%
	9∼12 days	2385	62.27%
	Total	3830	100.00%

### IVIG non-responsiveness

Enzyme processing (beta-propriolactonation) increased the risk of non-responsiveness significantly, with a relative risk of 1.45 (95% CI 1.08∼1.94). Although acidification had a relative risk of 0.79, it did not reach statistical significance (95% CI 0.61∼1.01). IgA depletion had no significant impact on the primary outcome (Table 3).

**Table 3:** Univariate analysis of the study endpoints (non-responsiveness to IVIG).

		n	Treatment failure	rate	RR	95%CI		
β-propiolactonation	Yes	617	52	8.43%	1.45	1.08	∼	1.94
	No	3213	187	5.82%				
Acidification	Yes	1894	104	5.49%	0.79	0.61	∼	1.01
	No	1936	135	6.97%				
Containing IgA	Yes	2511	156	6.21%	0.99	0.76	∼	1.28
	No	1319	83	6.29%				

		n	acute aneurysm	rate	RR	95%CI		
β-propiolactonation	Yes	617	30	4.86%	0.70	0.48	∼	1.02
	No	3213	223	6.94%				
Acidification	Yes	1894	150	7.92%	1.49	1.17	∼	1.90
	No	1936	103	5.32%				
Containing IgA	Yes	2511	180	7.17%	1.30	1.00	∼	1.69
	No	1319	73	5.53%				

		n	prolonged use of anti-platelet or anti-coagulant	rate	RR	95%CI		
β-propiolactonation	Yes	617	101	16.37%	1.44	1.18	∼	1.76
	No	3213	365	11.36%				
Acidification	Yes	1894	207	10.93%	0.82	0.69	∼	0.97
	No	1936	259	13.38%				
Containing IgA	Yes	2511	308	12.27%	1.02	0.86	∼	1.23
	No	1319	153	11.60%				

CI: confidence interval, RR: relative risk.

### Acute aneurysm

Enzyme processing (beta-propriolactonation) had a relative risk of 0.70, but it did not reach statistical significance. Acidification increased the risk of aneurysm with a relative risk of 1.30 (95% CI 1.17∼1.90). Although IgA depletion had a relative risk of 1.30, it did not reach statistical significance (Table 3).

### Prolonged use of anti-platelets or anti-coagulants

Enzyme processing (beta-propriolactonation) increased the risk of prolonged use of anti-platelets or anti-coagulants, with a relative risk of 1.44 (95% CI 1.18∼1.76). Acidification had a protective effect with a relative risk of 0.82 (95% CI 0.69∼0.97). IgA depletion had no significant impact on the primary outcome (Table 3).

### Recurrence

**Figure 2:**
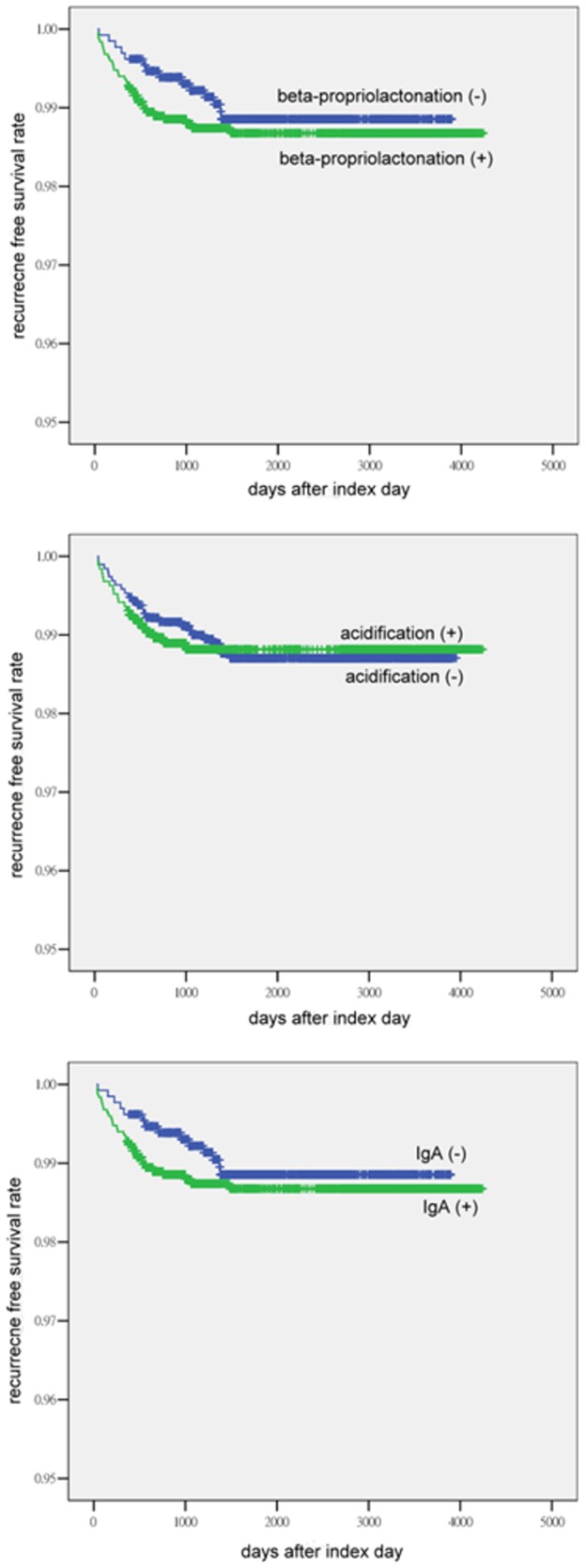
Recurrence-free survival curves according to the different manufacturing processes.

In the analysis of recurrence-free survival, neither acidification nor IgA depletion showed a significant impact on recurrence. Although separation of the curves was observed when grouped by enzyme processing (beta-propriolactonation), it did not reach statistical significance. This may have been due to a limit of statistical power (Figure 2).

### Multiple logistic regression models

We used multiple logistic regression models to investigate whether cofactors influenced the crude rate estimation as described above. For non-responsiveness, the adjusted rates were not significantly different from the crude rates (Table 4). For acute aneurysms, the direction of the odds ratio did not change, however the protective effect of beta-propriolactonation became more significant (Table 4). In prolonged use of anti-platelets or anti-coagulants, beta-propriolactonation still increased the risk when adjusting for the cofactors. However, the protective effect of acidification became non-significant (Table 4).

**Table 4:** Results of multiple logistic regression.

IVIG non-responsiveness					
		OR	95% CI		
*β*-propiolactonation	crude	1.49	1.08	∼	2.05
	model 1	1.44	1.04	∼	2.00
	model 2	1.52	1.01	∼	2.27
Acidification	crude	0.78	0.60	∼	1.01
	model 1	0.76	0.59	∼	1.00
	model 2	0.65	0.47	∼	0.88
Containing IgA	crude	0.99	0.75	∼	1.30
	model 1	0.94	0.71	∼	1.25
	model 2	0.78	0.56	∼	1.09
**Acute aneurysms**					
*β*-propiolactonation	crude	0.69	0.46	∼	1.01
	model 1	0.53	0.36	∼	0.78
	model 2	0.39	0.25	∼	0.61
Acidification	crude	1.53	1.18	∼	1.98
	model 1	1.38	1.06	∼	1.80
	model 2	1.50	1.10	∼	2.05
Containing IgA	crude	1.32	1.00	∼	1.75
	model 1	0.98	0.74	∼	1.31
	model 2	0.85	0.60	∼	1.22
**Prolonged use of anti-platelet or anti-coagulants**					
*β*-propiolactonation	crude	1.53	1.20	∼	1.94
	model 1	1.49	1.16	∼	1.90
	model 2	1.42	1.06	∼	1.89
Acidification	crude	0.79	0.65	∼	0.97
	model 1	0.77	0.63	∼	0.94
	model 2	0.85	0.68	∼	1.06
Containing IgA	crude	1.03	0.84	∼	1.26
	model 1	0.97	0.78	∼	1.19
	model 2	1.05	0.82	∼	1.35

CI: confidence interval, OR: odds ratio.

Model 1: adjusting for fever days, hospital level, gender.

Model 2: adjusting for fever days, hospital level, gender, congenital heart disease, period (years), and age <1 year.

### Sensitivity analysis for primary outcome

Because we arbitrarily defined IVIG non-responsiveness as the total amount exceeding 30 gm in one admission, the endpoint may have been subject to misclassification. Therefore, we performed sensitivity analysis. When we changed the cut-off point from a total dose of 24 gm to 38 gm, there was no significant change in endpoint estimation (Table 5).

**Table 5:** Sensitivity analysis for odds ratios for treatment failure by β-propriolactation, acidified and IgA depletion.

	Treatment failure No.						Treatment failure No.						Treatment failure No.					
Cutoff Points (gm)	Propio (+) N = 617	Propio (−) N = 3213	OR	95% CI			Acidified (+) N = 1864	Acidified (−) N = 1936	OR	95% CI			IgA (+) N = 2511	IgA (−) N = 1319	OR	95% CI		
24	600	125	1.10	0.88	∼	1.36	362	363	1.04	0.88	∼	1.22	237	488	1.10	0.93	∼	1.31
26	299	73	1.30	0.99	∼	1.71	208	164	0.79	0.64	∼	0.98	135	237	0.92	0.74	∼	1.15
28	254	62	1.30	0.97	∼	1.74	177	139	0.79	0.63	∼	1.00	115	201	0.92	0.72	∼	1.17
29	252	62	1.31	0.97	∼	1.75	177	137	0.78	0.62	∼	0.99	115	199	0.91	0.71	∼	1.15
30	187	52	1.48	1.07	∼	2.04	135	104	0.79	0.60	∼	1.02	83	156	0.99	0.75	∼	1.31
31	186	52	1.49	1.08	∼	2.05	135	103	0.78	0.60	∼	1.01	83	155	0.99	0.75	∼	1.30
32	186	52	1.49	1.08	∼	2.05	135	103	0.78	0.60	∼	1.01	83	155	0.99	0.75	∼	1.30
34	179	50	1.48	1.07	∼	2.06	130	99	0.78	0.59	∼	1.02	80	149	0.98	0.74	∼	1.31
36	154	46	1.59	1.13	∼	2.24	114	86	0.77	0.58	∼	1.03	68	132	1.03	0.76	∼	1.39
38	142	43	1.61	1.12	∼	2.29	110	75	0.70	0.51	∼	0.94	67	118	0.93	0.68	∼	1.27

CI: confidence interval; OR: odds ratio; Propio: β-propriolactation.

## Discussion

This is a nationwide cohort study with longitudinal follow-up to evaluate the comparative effectiveness of IVIG for Kawasaki disease. This is the first longitudinal study to evaluate the effectiveness of immunoglobulin, and also includes the largest number of cases of any study on this issue.

We found that beta-propriolactonation increased the risk of IVIG non-responsiveness. In contrast, acidification decreased the risk after adjusting for cofactors. These findings are consistent with a previous report by Tsai and colleagues [28]. A possible explanation for this is that the therapeutic effect of IVIG in treating Kawasaki disease may come from immune modulatory effects by the constant region (Fc portion) of immunoglobulin [21]. It has been reported that IVIG decreases interleukin-1 release from monocytes without decreasing the level of cytotoxic anti-endothelial antibodies [32]. It has also been reported that interleukin-1 release is suppressed by the interaction between the Fc portion of immunoglobulin and the Fc receptors of macrophages [33]. Because amino acid residues are changed during alkylation and acylation of proteins during beta-propriolactonation, the Fc portion of immunoglobulin may be modified more in comparison with ion exchange or diafiltration. As a result, enzyme processing may lead to poorer immunomodulation function by an interaction between the Fc portion and the Fc receptors on immune cells [34], [35]. This may explain why beta-propriolactonation had the poorer clinical outcomes.

With regards to acidification, Manlhiot and colleagues reported similar observations, in that acidification reduced the risk of IVIG non-responsiveness. The authors attributed the mechanism to differences in IVIG concentration (10% vs. 5%), because a higher concentration of IVIG needs a shorter infusion time, and rapid infusion may lead to better clinical outcomes [26]. In Taiwan, most physicians follow the guidelines of the AHA and AAP [21], which recommend rapid infusion in 12 hours. So, we do not think that a difference in infusion time can explain the phenomenon we observed in Taiwan.

Acidification increased the risk of coronary aneurysms, which is consistent with the observations by Manlhiot and colleagues [26]. This may be because acute inflammation in Kawasaki disease leads to the damage of elastin, and as a result weakens the blood vessels. It is currently thought that a large amount of acidic fluid infusion may lead to a decrease in intracellular tone and increase in vascular tension [36]–[38]. This may explain the observed increase in acute coronary aneurysms.

For prolonged use of anti-platelets or anti-coagulants, neither Tsai et al. nor Manlhiot et al. provided long-term follow-up information [26], [28]. In our study, the direction of points of estimation were similar with those of non-responsiveness to IVIG. This is reasonable because rapid suppression of immune responses means less coronary artery damage and faster normalization of laboratory data, both of which could possibly decrease the need for prolonged use of mediation.

For recurrence, no significant differences were found among all the methods of preparation, although a separation of the curves was observed with beta-propriolactonation. This may have been caused by a limitation in statistical power. If there had been a larger number of patients, statistical significance may have been reached.

Because only the total amount of IVIG was available in the claims data and there were no therapeutic orders for IVIG infusion, the primary endpoint of this study was arbitrarily defined as a total dose exceeding 30 gm, and therefore misclassification was possible. We thus performed sensitivity analysis, and found that the odds ratio did not change significantly when changing the cut-off point from a total dose of 24 gm to 38 gm (Table 5). Furthermore, we performed a secondary analysis by limiting the range of the patients' age from 6 months to 2 years old (Table 6). The point estimations and confidence intervals were also very close to those in Table 5. From these two tests, it is reasonable to assume that the primary endpoint estimation in this study is robust despite a certain degree of outcome misclassification. For children older than 2 years, we considered that the misclassification would become worse because the variability in body weight increases, and they were therefore excluded.

**Table 6:** Univariate analysis after limiting the age from 6 months to 2 years.

		n	Treatment failure	rate	RR	95%CI		
β-propiolactonation	Yes	488	46	9.43%	1.47	1.08	∼	2.01
	No	2574	165	6.41%				
Acidification	Yes	1498	93	6.21%	0.82	0.63	∼	1.07
	No	1564	118	7.54%				
Containing IgA	Yes	1986	139	7.00%	1.05	0.79	∼	1.38
	No	1076	72	6.69%				

		n	Acute aneurysm	rate	RR	95%CI		
β-propiolactonation	Yes	488	25	5.12%	0.73	0.49	∼	1.09
	No	2574	181	7.03%				
Acidification	Yes	1498	120	8.01%	1.46	1.11	∼	1.90
	No	1564	86	5.50%				
Containing IgA	Yes	1986	145	7.30%	1.29	0.96	∼	1.72
	No	1076	61	5.67%				

		n	Prolonged use of anti-platelets or anti-coagulants	rate	RR	95%CI		
β-propiolactonation	Yes	488	74	15.16%	1.47	1.16	∼	1.35
	No	2574	266	10.33%				
Acidification	Yes	1498	153	10.21%	0.85	0.70	∼	1.04
	No	1564	187	11.96%				
Containing IgA	Yes	1986	227	11.43%	1.09	0.88	∼	1.35
	No	1076	113	10.50%				

CI: confidence interval, RR: relative risk.

This is an observational study, and confounders may have existed. Therefore we used two multiple logistic regression models to examine whether the point estimates were changed by cofactors (Table 4). However, the odds ratio estimations were similar between the adjusted and crude rates.

The definition of acute aneurysms by ICD9-CM code alone may not be very accurate. However, in order to maintain comparability with previous research [3], we used the same definition. In this study, the rate of acute coronary aneurysm (6.61%) is very close to Huang's estimation in Taiwan [3] but lower than the 9.3% reported in Japan [1] and 19.8% in Shanghai, China [39]. Because the patients' identification information was scrambled before data release, we could not identify the real reasons by validating the data. However, the rate of coronary aneurysm may have been be underestimated because the physicians may not have coded if they considered that the coronary anomalies were mild. With regards to chronic coronary aneurysms, we initially used ICD9-CM codes in the outpatient records, however, only 19 cases were defined as such. As a result, instead of ICD9-CM codes, we chose to define chronic coronary aneurysms by prescriptions of anti-platelet or anti-coagulation medication in outpatient visits. Nevertheless, the rate was higher than expected, and we don't think it represents the true rate of chronic aneurysms. Physicians may prescribe medications for laboratory anomalies according to the AAP and AHA guidelines [21]. Although the prescriptions of anti-platelets or anti-coagulants may not fulfill the guidelines, it reflects the real clinical practice of physicians who care for children with Kawasaki disease.

The aim of this study was to compare the effectiveness of IVIG produced by different manufacturing processes, and the patients who did not receive IVIG therapy were not our target study population. Taiwan has the third high incidence of Kawasaki disease worldwide, so pediatricians in Taiwan are familiar with diagnosing Kawasaki disease. If a child was coded as having Kawasaki disease and received IVIG, the possibility of misdiagnosis should therefore be low. Atypical Kawasaki disease is not defined in the claims data. However, the treatment should be the same because the patients with atypical Kawasaki disease are also prone to coronary complications [40]. Moreover, the NHI of Taiwan covers the fee for the treatment. Therefore, we do not think that differentiating atypical from typical Kawasaki disease is a critical issue for this study.

Genome predisposition to the susceptibility and complications of Kawasaki disease has been reported in Taiwan, especially the ITKPC gene polymorphism [41]–[43]. If the difference in comparative effectiveness of IVIG can be proven to be associated with a specific genome polymorphism, it would be very helpful in developing personalized medicine.

The NHIRD covers almost the entire population of Taiwan. However, this study still has some limitations. First, the patients' identification numbers were scrambled making it impossible to validate the data. Second, the severity of coronary aneurysm could not be identified solely from the diagnosis codes, making it necessary to use surrogate endpoints such as use of anti-coagulants to define persistent aneurysms. Third, the NHIRD lacks laboratory data, thereby limiting the analysis of some important cofactors of disease outcomes [44], [45].

## Conclusions

In summary, beta-propriolactonation of IVIG may lead to a higher risk of IVIG non-responsiveness and prolonged use of anti-platelets or anti-coagulants in the treatment of Kawasaki disease, and acidification of IVIG may be associated with a higher acute coronary aneurysm rate. Further larger scale and prospective studies are needed to elucidate this issue.
